# Supplementary material for: Conformational dynamics during misincorporation and mismatch extension defined using a DNA polymerase with a fluorescent artificial amino acid
Source: J Biol Chem. 2021 Nov 25;298(1):101451. doi: 10.1016/j.jbc.2021.101451 (PMC8715121; doi:10.1016/j.jbc.2021.101451)
Supplement: Figure S1 [file mmc1.pdf]

Supplemental information for:

Conformational dynamics during misincorporation and mismatch extension defined using a DNA polymerase with a fluorescent artificial amino acid.

by

Tyler L. Dangerfield, Serdal Kirmizialtin, and Kenneth A. Johnson

This file contains the supplemental figure described in the main text.

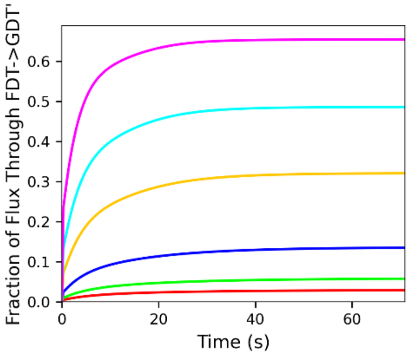

Figure S1: Flux through activated GDT' state. A simulation in KinTek Explorer was performed using the same experimental conditions as the experiment described in Figure 4A and the fractional flux of the FDT state through the GDT' state versus the total flux through GDT + GDT'. At low nucleotide concentrations (up to 250 μM, blue trace), less than 10% of the FDT state goes through the GDT' pathway. At higher concentrations (3 mM, purple trace), greater than 60% goes through the activated GDT' pathway.
